# Epithelial-mesenchymal transition-related circular RNAs in lung carcinoma

**DOI:** 10.20892/j.issn.2095-3941.2020.0238

**Published:** 2021-06-15

**Authors:** Meina Jiang, Shuai Fang, Xiaodong Zhao, Chengwei Zhou, Zhaohui Gong

**Affiliations:** 1 Department of Biochemistry and Molecular Biology, Ningbo University School of Medicine, Ningbo 315211, China; 2 Zhejiang Province Key Laboratory of Pathophysiology, Ningbo University School of Medicine, Ningbo 315211, China; 3 Department of Thoracic Surgery, The Affiliated Hospital of Ningbo University School of Medicine, Ningbo 315020, China

**Keywords:** Epithelial-mesenchymal transition, circular RNA, transcription factor, metastasis, lung carcinoma

## Abstract

The epithelial-mesenchymal transition (EMT) is a highly complex phenotypic conversion during embryogenesis, and is important for metastasis, which contributes to tumor deterioration and poor prognoses of cancer patients. Lung carcinoma has a high tendency to develop the EMT. Circular RNAs (circRNAs) are involved in EMT-related cell invasion and metastasis in various types of cancers. Moreover, circRNAs have been found to be a link to EMT-related transcription factors and EMT-associated signaling pathways. This review mainly focuses on the influence of EMT-related circRNAs on lung carcinomas. More specifically, the roles of EMT-inducing- and EMT-suppressive circRNAs in lung carcinomas are discussed. With circRNAs potentially becoming promising biomarkers and therapeutic targets for cancer managements, they will hopefully stimulate the interest of medical workers in the early diagnosis, personalized treatment, and positive prognoses in the era of precision oncology.

## Introduction

The epithelial-mesenchymal transition (EMT) is a highly complicated phenotypic conversion during embryogenesis, and is a potential process in adults by which epithelial cells gradually downregulate the expressions of cytokeratins and E-cadherin, and upregulate the expressions of mesenchymal genes such as vimentin and fibronectin^1^. Generally, the EMT can be divided into three subtypes: type I EMT in embryonic development, type II EMT in fibrosis, and type III EMT in premalignant and malignant stroma^2^. It is worth noting that EMT is not a purely bipolar state with two well-defined cell populations of mesenchymal cells and epithelial cells. There is also an intermediate state, called partial, involving incomplete hybrid EMT states expressing various levels of epithelial and mesenchymal characteristics and preserving intermediate morphologies^3–5^. The EMT is involved in multiple tumor processes including tumor initiation, stemness, migration, cancerous progression, intravasation into the blood, malignant metastasis, and resistance to therapy^6^. However, EMT-inducing transcription factors (EMT-TFs), EMT-related signaling pathways, epigenetic controls, and post-transcriptional regulators have also been found to regulate the EMT^7^. Nonetheless, the roles of circular RNAs (circRNAs) as post-transcriptional regulators in modulating the EMT remain unclear.

With the rapid development of high-throughput RNA sequencing (RNA-seq), a variety of circRNAs have been characterized in humans. In the last few years, it has been reported that circRNAs possess EMT-associated functions and may have an effect on epithelial and mesenchymal cell characteristics including metastasis, migration, and invasion^8^. It is known that circRNAs are a type of covalent single chain closed-loop molecule lacking the 5′end cap and 3′end poly (A) tails *via* a form of alternative splicing, resulting in more stability than linear RNAs in the presence of RNase R^9^. The circRNAs can be divided into three classifications: exonic circRNAs (ecircRNAs), circular intronic RNAs (ciRNAs), and exon-intronic circRNAs (EIciRNAs). Based on their translational capabilities, circRNAs can be classified into noncoding circRNAs and protein-coding circRNAs^10^. In future studies, EMT-related circRNAs will therefore be extensively studied in the field of oncology.

With a 5-year survival rate of 16.6%, lung cancer is the most common cancer with two major categories of non-small cell lung cancer (NSCLC) and small cell lung cancer (SCLC), and is the leading cause of cancer-related deaths^11,12^. Despite great progress in experimental research and clinical treatments, lung carcinoma still has a poor diagnosis and prognosis. Thus, there is an urgent need to improve our understanding of the mechanisms of tumorigenesis in the lung^13^. Recently, studies have started to decipher the significance of EMT-related circRNAs in various carcinomas. Lung carcinoma tends to develop as a result of the EMT and metastasis; however, the function of EMT-related circRNAs is still unclear. This review focuses on EMT-related circRNAs in lung carcinoma. It is hoped that it will stimulate interest in the early diagnosis, personalized treatment, and positive prognoses in the field of precision oncology.

## Biosynthesis, characteristics, and functions of circRNAs

**Figure 1:**
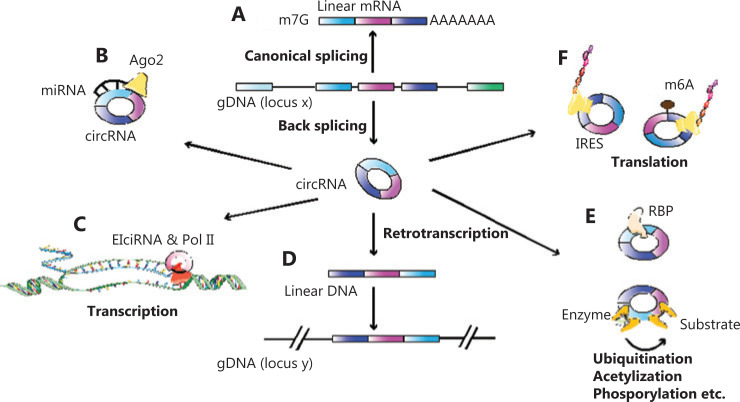
Biosynthesis and functions of circular RNAs (circRNAs). (A) Pre-mRNAs generate mRNAs through canonical splicing while circRNAs are generated through back-splicing. (B) CircRNAs bind to miRNAs. (C) Exon-intronic circRNAs bind to pol II to regulate transcription. (D) The circRNA-derived pseudogenes insert into the genome and switch the genomic DNA configuration through circRNA-retrotranspostion. (E) CircRNAs control protein-protein interactions in a similar manner to reservoirs or scaffoldings. (F) CircRNAs initiate translation through internal ribosome entry sites or *via* N6-methyladenosine.

In the past, numerous circRNAs have been found at lower levels of expression by using outdated detection technologies. As a result, they have been recognized as nonspecific byproducts^14^. However, it is now known that circRNAs play more critical roles with their unique biosynthesis and characteristics, when compared to cognate linear RNAs. Most circRNAs are created by back-splicing in pre-mRNA, of which 3′ splice donors covalently link to 5′ splice acceptors in the opposite direction (**Figure 1A**). The back-splicing includes four paradigms such as exon-skipping, lariat-driven, intron-pairing-driven circularization, and RNA binding protein-driven circulation^15^. Furthermore, circRNAs have four distinct peculiarities. First, they are conservative despite the length of their evolution^16^. Second, they are specifically expressed spatiotemporally in altered cell types and tissues^17^. Third, they are abundant in all species containing plants, archaea, mice, and humans^9,18–20^. Finally, they have a high stability because of their closed-loop construction and short free terminals. Although circRNAs are stable, the understanding of circRNA degeneration is still unclear. Some hypotheses suggest that endonucleases or N6-methyladenosine (m6A) may initiate degradation of circRNAs. Alternatively, circRNAs may be degraded when combined with miRNAs during Ago2-facilitated cleavage^21,22^.

Regarding biological functions, circRNAs play different roles in different subcellular locations. In the cytoplasm, circRNA segregates miRNA as a “sponge” to modulate the activity of miRNA and the miRNA-targeted gene (**Figure 1B**). For example, Zhang et al.^23^ reported that circFOXO3 (hsa_circ_0006404) promoted the expression of nuclear factor of activated T cells 5 (NFAT5) as a sponge of both miR-138-5p and miR-432-5p in glioblastoma tissues. In the nucleus, circRNA regulates the splicing process or gene transcription (**Figure 1C**). Huang et al.^24^ showed that circERBB2, a circRNA generated from the *ERBB2* cognate gene, accumulated in the nucleus to bind with PA2G4, a nucleolus-associated protein, to regulate ribosomal DNA transcription and cell proliferation *via* the circERBB2-PA2G4-TIFIA axis in gallbladder cancer. Like linear mRNAs, circRNAs can also generate pseudogenes. The circRNA-derived pseudogenes are capable of inserting into the genome and switching the genomic DNA configuration during circRNA retrotransposition^25^ (**Figure 1D**). Moreover, circRNAs, in a similar manner to reservoirs or scaffoldings, can bind proteins in exact subcellular positions to control protein-protein interactions^26^ (**Figure 1E**). Presently, it is well-known that circRNAs involve one of the spliced production coding peptides. For example, Ye et al.^27^ showed that circFBXW7 (hsa_circ_0001451) encoded a 185 amino acid peptide (FBXW7-185aa) inhibiting migration and proliferation in triple-negative breast cancer cells. Yet unlike canonic cap-dependent translations, circRNAs commence translation *via* the internal ribosome entry site (IRES) or through m6A^27,28^ (**Figure 1F**).

## The circRNA link to the EMT in multiple cancers

The EMT comprises fine-tuned phenotypic shifts, in which cells imperceptibly decline expressing E-cadherin and lose cellular adhesion and apical-basal polarity, while showing increased expressions of N-cadherin, extracellular matrix degradation, and cytoskeletal reorganization. Such shifts are initiated by EMT-TFs, which are regulated by EMT-related signaling pathways. EMT-inducing signs are spatiotemporally specific when interacting with numerous regulators and signaling pathways. Although the contributions of the EMT to drug resistance and chemotherapy tolerance have been well-studied, the EMT promotion of tumor metastasis is still controversial^29^. However, increasing evidence has suggested that circRNA-mediated EMT is an essential part of tumor occurrence and development (**Table 1**).

**Table 1:** CircRNAs modulating the epithelial-mesenchymal transition (EMT) in various carcinomas

Cancer type	CircRNA	Function	Mechanism	Reference
HCC	circCul2 (hsa_circ_10720)	Promoted EMT	Twist1 promoted vimentin expression and EMT by increasing levels of circCul2, which can absorb miRNAs that target vimentin	^31^
UCB	circPRMT5 (circRNA_101320)	Promoted EMT and aggressiveness	By circPRMT5/miR-30c/SNAIL1/E-cadherin pathway	^34^
Melanoma	circRNA_0084043	Promoted growth and metastasis	By circRNA_0084043/miR-153-3p/Snail axis	^35^
Cervical cancer	circRNA_000284	Promoted proliferation and cell invasion	By circRNA_000284/miR-506/Snail 2 axis	^36^
PTC	circNUP214 (hsa_ circ_0089153)	Promoted proliferation, migration and invasion	By circNUP214/miR-145/ZEB2 axis	^37^
TNBC	circANKS1B (hsa_circ_0007294)	Promoted EMT and metastasis	By circANKS1B/miR-152-3p, miR-148a-3p/USF1/TGF-β1/Smad signaling and a positive feedback loop of USF1/circANKS1B	^38^
OSCC	circUHRF1 (hsa_circ_0002185)	Promoted proliferation, metastasis, invasion and EMT	By a positive feedback loop of circUHRF1/miR-526b-5p/c-Myc/TGF-β1/ESRP1/circUHRF1	^39^
CRC	circRNA_100290	Promoted metastasis and EMT	By circRNA_100290/miR-516b/FZD4/Wnt/β-catenin signaling pathway	^40^
ccRCC	circAKT3	Inhibited metastasis and EMT	By circAKT3/miR-296-3p/E-cadherin pathway	^41^
DLBCL	circAPC (hsa_circ_0127621)	Hindered cell proliferation	By circAPC/APC/TET1/Wnt/β-catenin signaling pathway	^42^
CRC	hsa_circ_0026344	Functioned as anti-tumor molecule	By hsa_circ_0026344/miR-183/Wnt/β-catenin signaling pathway	^43^
Bladder cancer	circPTK2 (hsa_circ_0003221)	Enhanced migration	Unknown	^59^
CRC	circPTK2 (hsa_circ_0005273)	Stimulated EMT	Attaching to vimentin protein at sites Ser38, Ser55, Ser82	^60^

HCC, hepatocellular carcinoma; UCB, urothelial carcinoma of the bladder; PTC, papillary thyroid cancer; TNBC, triple-negative breast cancer; OSCC, oral squamous cell carcinoma; CRC, colorectal cancer; ccRCC, clear cell renal cell carcinoma; DLBCL, diffuse large B-cell lymphoma.

### The circRNA link to EMT-TFs

**Figure 2:**
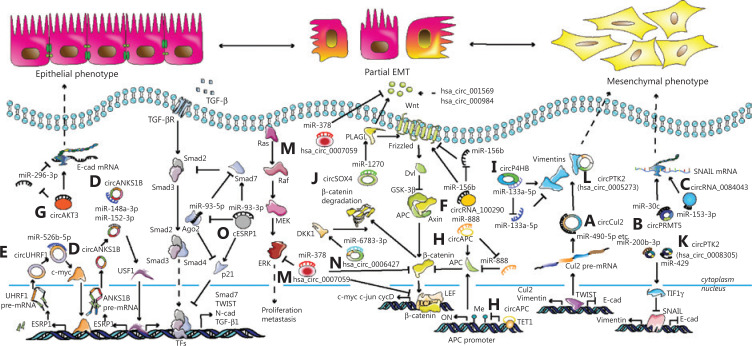
Mechanisms of the circRNA-mediated epithelial-mesenchymal transition (EMT) and metastasis in various carcinomas. (A) Twist promotes circCul2 expression by sponging miR-490-5p, and results in the upregulation of vimentin in hepatocellular carcinoma. (B) CircPRMT5 mediates the EMT in urothelial carcinoma of the bladder through the circPRMT5/miR-30c/SNAIL1 axis. (C) CicrRNA_0084043 increases metastasis in melanomas by regulating the miR-153-3p/Snail axis. (D) CircANKS1B facilitates tumor metastasis and invasion in triple-negative breast cancer through the circANKS1B/miR-148a-3p/miR-152-3p/USF1/TGF-β1 axis and a positive feedback loop of USF1/ESRP1/circANKS1B. (E) CircUHRF1 promotes migration, invasion, and the EMT in oral squamous cell carcinoma through a positive feedback loop of circUHRF1/miR-526b-5p/c-Myc/TGF-β1/ESRP1/circUHRF1. (F) CircRNA_100290 induces metastasis and the EMT in colorectal cancer (CRC) through the miR-516b/FZD4/Wnt/β-catenin signaling pathway. (G) CircAKT3 accelerates metastasis and the EMT in clear cell renal carcinoma by the circAKT3/miR-296-3p/E-cadherin axis. (H) CircAPC decreases the canonical Wnt/β-catenin signaling pathway in diffuse large B-cell lymphoma by the miR-888/APC axis in the cytoplasm and circAPC/TET1/APC in the nucleus. (I) CircP4HB promotes the EMT in non-small cell lung cancer (NSCLC) by the circP4HB/miR-133a-5p/vimentin axis. (J) CircSOX4 facilitates the EMT in lung adenocarcinoma (LUAD) by the circSOX4/miR-1270/PLAGL2 axis. (K) CircPTK2 (hsa_circ_0008305) enhances the EMT in NSCLC by the circPTK2/miR-429/miR-200b-3p/TIF1γ/snail axis. (L) CircPTK2 (hsa_circ_005273) stimulates the EMT in CRC *via* directly combining with vimentin. (M) Hsa_circ_0007059 inhibits the EMT, decreases Wnt/β-catenin, and ERK1/2 signaling pathways in A549 and H 1975 cells by suppressing miR-378. (N) Hsa_circ_0006427 represses the Wnt/β-catenin signaling pathway in LUAD *via* the miR-6783-3p/DKK1 axis. (O) cESRP1 suppresses the EMT in NSCLC *via* the cESRP1/miR-93-5p/Smad7/p21 axis.

Vimentin is a type of EMT protein marker, which is present in mesenchymal cells and is involved in cancer metastasis and poor prognoses. Cullin2 (Cul2), as a tumor suppressor protein, is a principal part of the multiple cullin-RING-based ECS (Elongin B/C-Cul2/5-SOCS-box protein) E3 ubiquitin-protein ligase complexes engaging in cell cycle control and vasculogenesis^30^. Twist, a vital EMT-TF, combines with the promoter of Cul2 to increase the expression of circCul2 (hsa_circ_10720), which binds to a set of miRNAs to increase vimentin expression in hepatocellular carcinoma (HCC)^31^ (**Figure 2A**). In addition to Twist, the EMT is also initiated by Snail, which is a conservative protein of the zinc finger transcription factor family, containing zinc finger domains to bind DNA at the C-terminus, and containing the SNAG domain to interact with epigenetic remodeling complexes at the N-terminus^32,33^. In urothelial carcinoma of the bladder (UCB), circPRMT5 (has_circRNA_101320) generated from *PRMT5* on chromosome 14q11.2, promotes the EMT and aggressiveness through the circPRMT5/miR-30c/SNAIL1/E-cadherin pathway^34^ (**Figure 2B**). In melanomas, circRNA_0084043 contributes to cell metastasis and growth *via* the miR-153-3p/snail axis^35^ (**Figure 2C**). In cervical cancer, circRNA_000284 induces cell invasion and proliferation *via* the circRNA-000284/miR-506/Snail-2 axis^36^. Apart from Twist and Snail, ZEB2 has been associated with epithelial polarities and diverse malignancies. Li et al.^37^ found that circNUP214 (hsa_ circ_0089153), as an oncogenic molecule, sponged miR-145 to upregulate the expression of ZEB2 and thereby promote cell proliferation, migration, and invasion in papillary thyroid cancer (PTC). Taken together, the results have shown that circRNAs can bind the classic EMT-TFs to modulate the EMT process and cancer development.

### The circRNA link to EMT-related signaling pathways

In addition to the EMT-TFs, EMT-related signaling pathways had been reported to be linked to cancer-associated circRNAs. In triple-negative breast cancer (TNBC), circANKS1B (hsa_circ_0007294), derived from exons 5–8 of the *ANKS1B* gene, is an independent risk factor of the overall survival of patients with TNBC, because of the promotion of tumor metastasis and invasion. Mechanistically, circANKS1B segregates miR-152-3p and miR-148a-3p, and upregulates the expression of transcription factor, USF1, which increases the expression of TGF-β1 by binding with the TGF-β1 promoter, and then initiating TGF-β1/Smad signaling to stimulate the EMT. In addition, there is a positive feedback loop where USF1 upregulates circANKS1B expression by regulating the splicing factor ESRP1^38^ (**Figure 2D**). In the oral squamous cell carcinoma (OSCC), circUHRF1 (hsa_circ_0002185) is in excess and promotes migration, invasion, proliferation, and the EMT *in vitro* and *in vivo* *via* a positive feedback loop pathway of circUHRF1/miR-526b-5p/c-Myc/TGF-β1/ESRP1/circUHRF1^39^ (**Figure 2E**). In colorectal cancer (CRC), circRNA_100290 acts as an oncogene to increase metastasis and the EMT by sponging miR-516b, upregulating FZD4 expression, and then initiating the FZD4-induced Wnt/β-catenin signaling pathway^40^ (**Figure 2F**). Other than pro-oncogenic circRNAs, investigators have also discovered some EMT suppressive circRNAs. For example, in clear cell renal cell carcinoma (ccRCC), overexpression of circAKT3 decreases the EMT and metastasis *via* the circAKT3/miR-296-3p/E-cadherin pathway^41^ (**Figure 2G**). In diffuse large B-cell lymphoma (DLBCL), circAPC (hsa_circ_0127621), derived from the *APC* exon 7 to exon 14, decreases cell proliferation. CircAPC binds the DNA demethylase, TET1, and binds to the APC promoter to enhance the expression of APC, which decreases the Wnt/β-catenin signaling pathway in the nucleus. In addition, circAPC acts as a sponge of miR-888 to upregulate APC expression in the cytoplasm^42^ (**Figure 2H**). Notably, the EMT and metastasis induced by CCL20 and CXCL8 treatment are decreased by overexpression of anti-tumor hsa_circ_0026344, which binds to miR-183 and inhibits the Wnt/-catenin pathway^43^. In general, circRNAs modulate EMT and migration mainly through two signaling pathways: the TGF-β1 signaling and Wnt/β-catenin signaling pathways.

## The influence of EMT-related circRNAs on lung carcinoma

The EMT phenotype is commonly expressed in primary squamous cell carcinomas (SCCs) and lung adenocarcinomas (LUADs), and occurs early in the pathogenesis of SCC, suggesting a potential target for lung cancer chemoprevention and treatment^44^. In addition, the overexpression of forkhead box Q1 (FoxQ1) influences the poor prognosis in NSCLC and is associated with the EMT^45^. Further studies showed that the cancer stemness marker was associated with the EMT and predicted poor prognoses in patients with LUAD^46^. Thus, lung carcinoma has been shown to be linked to the EMT and metastasis^47^, and the EMT-related circRNAs have an effect on lung carcinomas (**Table 2**). Overall, the EMT-inducing and EMT-suppressive circRNAs play very important roles in the occurrence and development of lung carcinomas.

**Table 2:** The epithelial-mesenchymal transition (EMT)-related circRNAs in lung carcinoma

Type	CircRNA	Target/pathway
EMT-inducing circRNAs in NSCLC	circ-ENO1	circ-ENO1/miR-223p/ENO1 axis
	circAGFG1	circAGFG1/miR-203/ZNF281 axis
	circP4HB	circP4HB/miR-133a-5p/vimentin pathway
	hsa_circ_0007534	Unknown
	hsa_circ_0079530	Unknown
	circ_0067934	Unknown
	circRNA CCDC66	HGF and c-Met upregulate circRNA CCDC66, nAchRα7 downregulates circRNA CCDDC 66
	circ_0012673	circ_0012673/miR-320A/LIMK1 axis
	hsa_circ_000984	Wnt/β-catenin pathway
	circ_001569	transcription factor 4 and Wnt/β-catenin pathway
	circ-SOX4 (hsa_circ_0131457)	circ-SOX4/miR-1270/PLAGL2/Wnt signaling pathway
EMT-suppressive circRNAs in NSCLC	circPTK2 (hsa_circ_0008305)	circPTK2/miR-429/miR-200b-3p/Snail axis
	circPTPRA (hsa_circ_102984)	circPTPRA/miR-96-5p/RASSF8/E-cadherin
	hsa_circ_0007059	hsa_circ_0007059/miR-378/Wnt/β-catenin pathway and ERK1/2 pathway
	circ_0006427	circ_0006427/miR-6783e3p/DKK1/Wnt/β-catenin pathway
EMT-suppressive circRNAs in SCLC	cESRP1	cESRP1/miR-93-5p/Smad7/p21(CDKN1A) axis

ENO1, enolase 1; AGFG1, ArfGAP with FG repeats 1; ZNF281, zinc finger protein 281; P4HB, prolyl 4-hydroxylase subunit beta; CCDC66, coiled-coil domain containing 66; HGF, hepatocyte growth factor; nAchRα7, nicotinic acetylcholine receptor Alpha 7; LIMK1, LIM domain kinase 1; SOX4, SRY-box transcription factor 4; PTK2, protein tyrosine kinase 2; PTPRA, protein tyrosine phosphatase receptor type A; RASSF8, Ras association domain family member 8; DKK1, Dickkopf WNT signaling pathway inhibitor 1; EMT, epithelial mesenchymal transition; NSCLC, non-small cell lung cancer.

### The EMT-inducing circRNAs in NSCLC

Among the most common histological subtypes of lung carcinoma, LUAD is responsible for a majority of cancer-related mortalities worldwide. Zhou et al.^12^ reported that circENO1 and its host gene, *ENO1* were both amplified in LUAD cells to augment glycolysis and tumor growth, and silenced circENO1 impeded glycolysis, migration and the EMT, and induced cell apoptosis *via* the circENO1/miR-223p/ENO1 axis. Similarly, circAGFG1 increased the EMT of NSCLC cells *via* invasion and migration by acting as a sponge for miR-203, which targeted ZNF281^48^. In addition, Wang et al.^49^ reported that circP4HB was higher in NSCLC tissues than in healthy paired samples, and that circP4HB promoted higher vimentin expression and the EMT *in vivo* and *in vitro* *via* the circP4HB/miR-133a-5p/vimentin axis (**Figure 2I**). Qi et al.^50^ found that EMT-inducing circDDX42 (hsa_circ_0007534), a transcription product of DEAD-box helicase 42, decreased E-cadherin and increased the levels of Snail, N-cadherin, and vimentin. CircTwist1 (hsa_circ_0079530), which is 664 nt in length, was found to be upregulated in NSCLC. Knockdown of CircTwist1 resulted in the downregulation of mesenchymal marker proteins and the upregulation of epithelial marker proteins in A549 and H1299 cells^51^. Similarly, hsa_circ_0067934 led to an identical trend of changes of N-cadherin and vimentin, and an opposite effect for E-cadherin^52^. In LUAD, it was found that circCCDC66 and SUMO-activating enzyme subunit 2 (SAE2) were both highly expressed and associated with the EMT, lung cancer metastasis, and EGFR drug resistance. Hepatocyte growth factor (HGF) and c-Met upregulate SAE2 and circCCDC66 to enhance EMT and drug resistance; however, nicotinic acetylcholine receptor alpha 7 (nAChRα7) negatively modulates circCCDC66 expression^53^. LIM domain kinase 1 (LIMK1) is a serine-threonine protein kinase, which affects the actin cytoskeleton and participates in the EMT. Qin et al.^54^ showed that hsa_circ_0012673 upregulated LIMK1 *via* binding to miR-320a to manipulate migration, invasion, proliferation, apoptosis, and the EMT in lung cancer cells. Because the EMT is closely correlated with the Wnt-associated pathways, many studies have focused on the effects of circRNAs on the expression levels of cyclin D1, β-catenin, and c-myc in the Wnt/β-catenin signaling pathway. Li et al.^55^ reported that hsa_circ_000984 was highly expressed in NSCLC tissues and cells, and correlated with shorter disease-free survival and overall survival. Functionally, hsa_circ_000984 promoted EMT to a pro-oncogenic role *via* upregulating the expression of cyclin D1, β-catenin, and c-myc in the Wnt/β-catenin pathway. It was also reported that hsa_circ_001569 predicted a poor prognosis and enhanced the expressions of crucial members of the Wnt/β-catenin pathway, including Wnt1, β-catenin, and transcription factor 4 (TF4)^56^. In the same manner, upregulated circSOX4 (hsa_circ_0131457) activates the Wnt signaling pathway and promotes the EMT *via* the circSOX4/miR-1270/PLAGL2 axis in LUAD^57^ (**Figure 2J**). Taken together, it is clear that the EMT-inducing circRNAs promote EMT-mediated metastasis, mainly by affecting the major members of the EMT-related signaling pathways in NSCLC.

### The EMT-suppressive circRNAs in NSCLC

In contrast to EMT-inducing circRNAs, EMT-suppressive circRNAs usually negatively modulate EMT and inhibit certain cell programs such as migration, metastasis, and invasion. As mentioned above, Snail is one of the classical EMT-TFs. In NSCLC, circPTK2 (hsa_circ_0008305) binds to miR-429/miR-200b-3p to enhance transcriptional intermediary factor 1γ (TIF1γ), which suppresses the function of Snail in the nucleus^58^ (**Figure 2K**). Notably, circPTK2 (hsa_circ_0003221), derived from the *PTK2*, enhances migration in bladder cancer cells^59^. Recently, Yang et al.^60^ reported that circPTK2 (hsa_circ_0005273), unlike hsa_circ_0008305 in NSCLC, stimulated the EMT *via* binding to vimentin protein at Ser38, Ser55, and Ser82 in CRC (**Figure 2L**). Ras association domain-containing protein 8 (RASSF8) is an acknowledged tumor suppressor of lung cancer^61^. Wei et al.^62^ showed that circPTPRA (hsa_circ_102984) derived from protein tyrosine phosphatase receptor type A gene (*PTPRA*) inhibited tumor metastasis and the EMT by sponging miR-96-5p and releasing RASSF8 and E-cadherin. In A549 and H1975 cells, overexpression of hsa_circ_0007059 restrains cell proliferation, decreases the EMT, and hinders the Wnt/β-catenin and ERK1/2 signaling pathways *via* suppressing miR-378^63^ (**Figure 2M**). In addition, Yao et al.^64^ have shown that the cytoplasmic-located has_circ_0006427 sponges miR-6783-3p to release Dickkopf WNT Signaling Pathway Inhibitor 1 (DKK1) and inhibit the Wnt/β-catenin pathway, and thereby repress cell proliferation, migration, invasion and EMT in LUAD cells (**Figure 2N**). In summary, the EMT-suppressive circRNAs play important roles in suppressing EMT-mediated metastasis in NSCLC *via* acting as sponges of miRNAs to influence EMT-TFs, EMT-related signaling, and EMT markers.

### The EMT-suppressive circRNAs in SCLC

The effects of EMT-related circRNAs on SCLC presently remain largely unknown. Huang et al.^65^ showed that cESRP1 (circular RNA epithelial splicing regulatory protein 1) located in the cytoplasm sequestered miR-93-5p to free Smad7/p21(CDKN1A) and further regulate the TGF-β-induced EMT (**Figure 2O**). Moreover, the inhibition of the TGF-β pathway and cESRP1 overexpression improved the responsiveness to chemotherapy in a patient-derived xenograft model suffering acquired chemoresistance.

## Conclusions

Although numerous studies have verified that circRNAs are more stable than their cognate mRNAs, the effectiveness of back splicing is lower than canonical splicing from certain expression vectors^66^. Recent advances have shown that circRNAs can act as minimally-invasive or noninvasive biomarkers for various diseases including cancers, although the detection techniques in blood and other body fluids still need to be improved^15,67^. More importantly, circRNAs may work together as a group, with complicated crosstalk with other regulators^68^. The EMT is a chief element of the metastatic cascade, which is important in tumor deterioration. Additionally, the EMT is responsible for chemoresistance and immune resistance^69,70^. Although the correlations between miRNAs and EMT-TFs have been well-established^71^, as sponges for miRNAs, the roles of circRNAs in the EMT remain to be discovered. The levels of certain circRNAs could be distinctly changed during the EMT due to splicing programs stimulated by a set of splicing factors such as RBFOX2, SRSF2, and QKI^8^. CircRNAs function as parts of a noncoding RNA adjustment net by separating proteins and miRNAs during the EMT. A quantitative EMT scoring system based on gene expression profiles has recently been established to grade the status of the EMT^47^. This system has been used to emphasize the developmental lineage of each cancer subtype from microscopic and macroscopic EMT gradients, and each cancer subtype has a unique tendency to exhibit different EMT states. Lung carcinoma displays higher EMT scores, which always forecast a poor prognosis^47^. Investigators of EMT-related circRNAs can therefore provide novel therapeutic approaches and tactics for patients with lung carcinoma. Specifically, a large range of small molecule agents for targeting characteristics with EMT in lung carcinoma have been developed in preclinical phases^72^. For example, bufalin, a Chinese medicine, impedes the migratory activity of A549 human lung cancer cells and the TGF-β-induced EMT^73^. However, there is controversy concerning the oligonucleotide chemistry that targets circRNAs, but further studies may bring new hope to patients with drug resistance.
